# Historical Patterns and Drivers of Spatial Changes in Recreational Fishing Activity in Puget Sound, Washington

**DOI:** 10.1371/journal.pone.0152190

**Published:** 2016-04-07

**Authors:** Anne H. Beaudreau, Emily J. Whitney

**Affiliations:** University of Alaska Fairbanks, School of Fisheries and Ocean Sciences, 17101 Point Lena Loop Road, Juneau, AK, 99801, United States of America; The University of Sydney, AUSTRALIA

## Abstract

Small-scale fisheries are the primary users of many coastal fish stocks; yet, spatial and temporal patterns of recreational and subsistence fishing in coastal marine ecosystems are poorly documented. Knowledge about the spatial distribution of fishing activities can inform place-based management that balances species conservation with opportunities for recreation and subsistence. We used a participatory mapping approach to document changes in spatial fishing patterns of 80 boat-based recreational anglers from 1950 to 2010 in Puget Sound, Washington, USA. Hand-drawn fishing areas for salmon, rockfishes, flatfishes, and crabs were digitized and analyzed in a Geographic Information System. We found that recreational fishing has spanned the majority of Puget Sound since the 1950s, with the heaviest use limited to small areas of central and northern Puget Sound. People are still fishing in the same places they were decades ago, with relatively little change in specific locations despite widespread declines in salmon and bottomfish populations during the second half of the 20^th^ century. While the location of core fishing areas remained consistent, the size of those areas and intensity of use changed over time. The size of fishing areas increased through the 2000s for salmon but declined after the 1970s and 1980s for rockfishes, flatfishes, and crabs. Our results suggest that the spatial extent of recreational bottomfishing increased after the 1960s, when the availability of motorized vessels and advanced fish-finding technologies allowed anglers to expand their scope beyond localized angling from piers and boathouses. Respondents offered a wide range of reasons for shifts in fishing areas over time, reflecting substantial individual variation in motivations and behaviors. Changes in fishing areas were most commonly attributed to changes in residence and declines in target species and least tied to fishery regulations, despite the implementation of at least 25 marine preserves since 1970.

## Introduction

Fishing is a major driver of change in coastal marine ecosystems. Overfishing of coastal species is widespread and, in some cases, has been occurring for centuries [1]. The cumulative effects of fishing on coastal ecosystems depend not only on the numbers and sizes of organisms removed, but also on the spatial distribution of fishing activities. Because fish are not targeted randomly, fishing can lead to changes in the spatial distribution of harvested populations and have localized impacts on population and community structure through targeted removal of particular species and sizes [2]. Additionally, the direct impact of mobile fishing gear on the seafloor can lead to changes in benthic communities [3]. Consequently, understanding where people fish is important for assessing the ecological impacts of fishing and designing effective spatial management strategies, especially for species with limited mobility, high site fidelity, and small home ranges.

Although more attention has been paid to the impacts of commercial fishing, there is growing recognition that small-scale recreational and artisanal fishing activities can put substantial pressure on marine and freshwater resources [4, 5, 6]. Recreational fisheries are the primary users of many coastal fish stocks in developed, temperate regions [7, 4]. In spite of this, the spatial distribution of recreational fishing activities in coastal marine ecosystems is poorly documented [8]. Logbooks and electronic monitoring have allowed for better spatial resolution of commercial fishing activities, e.g., [9]; however, similar data are typically lacking for recreational and other small-scale fisheries [10]. In the United States, recreational fisheries catch and effort data are typically collected through telephone or in-person angler surveys, e.g., [11]. Fishing locations are rarely documented, except for some charter fisheries that have mandatory logbook reporting requirements (e.g., Alaskan sport fishing guides and charters), but these data are often inaccessible to researchers due to confidentiality concerns. Though it has not been widely applied to U.S. recreational fisheries, participatory mapping is a promising approach for collecting spatial information on recreational fishing activities using in-person interviews with fishers to document fishing locations, e.g., [12]. Participatory mapping has been used to characterize changes in spatial distribution of commercial fisheries over time [13, 14]; map artisanal fishing locations and effort [15, 16, 17]; identify fishing hot spots [18, 10]; and document local ecological knowledge of fish habitat and spawning areas [19, 20].

Characterizing long-term patterns in use of coastal habitats by anglers requires more than a static picture of where people fish. Information on the temporal stability of spatial fishing patterns and factors affecting the dynamics of where people fish is also needed to identify areas of importance to anglers, assess cumulative impacts of fishing, and prioritize areas for protection. Recreational fishers are a diverse group, demonstrating a wide range of motivations, interests, and experience [21]. Individual variation in fishing behavior, including where people choose to fish, is shaped by a wide range of social, economic, ecological, and personal factors. For example, spatial behavior of anglers can be affected by the density of other fishers or territoriality [19, 15]. Individuals may select fishing areas based on travel cost and logistical or technological considerations (e.g., access to GPS and motorized vessels; [13, 22]). Furthermore, fishing locations may vary over time in response to changing distribution and abundance of harvested species (e.g., increasing depth or distance from shore; [15, 14]) or regulations that prohibit fishing in certain locations, such as no-take marine protected areas. Predicting how the spatial distribution of recreational fishers may vary in response to future socioeconomic, regulatory, and environmental change relies on knowledge of the factors that have affected their choice of fishing locations in the past.

We used a low-cost, participatory mapping approach to document changes in recreational fishing patterns and factors affecting where people fished from 1950 to 2010 in Puget Sound, a large urbanized estuary in western Washington State, USA. Puget Sound is the second largest estuary in the United States and has undergone dramatic changes over recent decades. The human population has increased nearly 8-fold over the last 100 years in the Puget Sound region [23]. Coastal development, fishing, pollution, and other pressures have led to population declines of many marine species. In 2010, three species of rockfish (*Sebastes* spp.) in Puget Sound were listed for federal protection under the Endangered Species Act (ESA). Because rockfishes show strong fidelity to rocky reef habitats and occupy small home ranges, some as small as 10 square meters [24], they are particularly vulnerable to localized depletion. Therefore, developing a recovery plan for ESA-listed rockfishes requires spatial information about rockfish distributions and fishing activities targeting rockfish and other species. Recreational anglers have been the dominant fishery sector targeting rockfish and other species in Puget Sound since the 1970s [25]; however, very little historical information exists on the spatial extent of recreational fishing. Following the rockfish ESA listing, a moratorium was placed on targeted harvest and retention of rockfish in Puget Sound and adjacent marine waters and fishing for bottomfish was limited to 120 feet or shallower to minimize mortality of incidentally caught rockfish [26]. With these restrictions on bottomfishing, interviews with anglers are now the primary means by which to document changes in the use of space by the Puget Sound recreational sector.

The primary objective of this study was to document the spatial footprint of recreational fisheries in Puget Sound, characterizing both changes in the use of space by individual anglers and the overall spatial extent of recreational fishing by 80 experienced anglers since the 1950s. We posed two hypotheses about changes in use of space by recreational anglers based on patterns observed in other small-scale, coastal fisheries, e.g., [13, 22]: (1) on average, the size of individual anglers’ fishing areas expanded over time, as individuals gained greater access to distant locations through the use of motorized boats and due to local depletion of target species nearshore; and (2) the core areas used by the surveyed group of recreational fishers as a whole have expanded since the 1950s. Secondly, we evaluated differences in the spatial distribution of fishing among major target species. The spatial extent of fishing reflects, to some degree, the underlying distributions of targeted species [27]. Therefore, we hypothesized that the extent of fishing areas would be smaller for relatively sedentary species, like rockfishes, compared to wider-ranging, more migratory species, like salmon. Finally, we identified factors affecting behavioral changes of anglers to provide a foundation for understanding how anglers will respond to future regulatory, social, economic, and ecological change. Specifically, our objective was to characterize anglers’ reasons for expanding, contracting, or shifting their fishing locations over time. We expected that anglers’ reported reasons for temporal changes in fishing areas would be related to personal, biological, and regulatory factors.

## Materials and Methods

### Documentation of fishing areas

**Fig 1:**
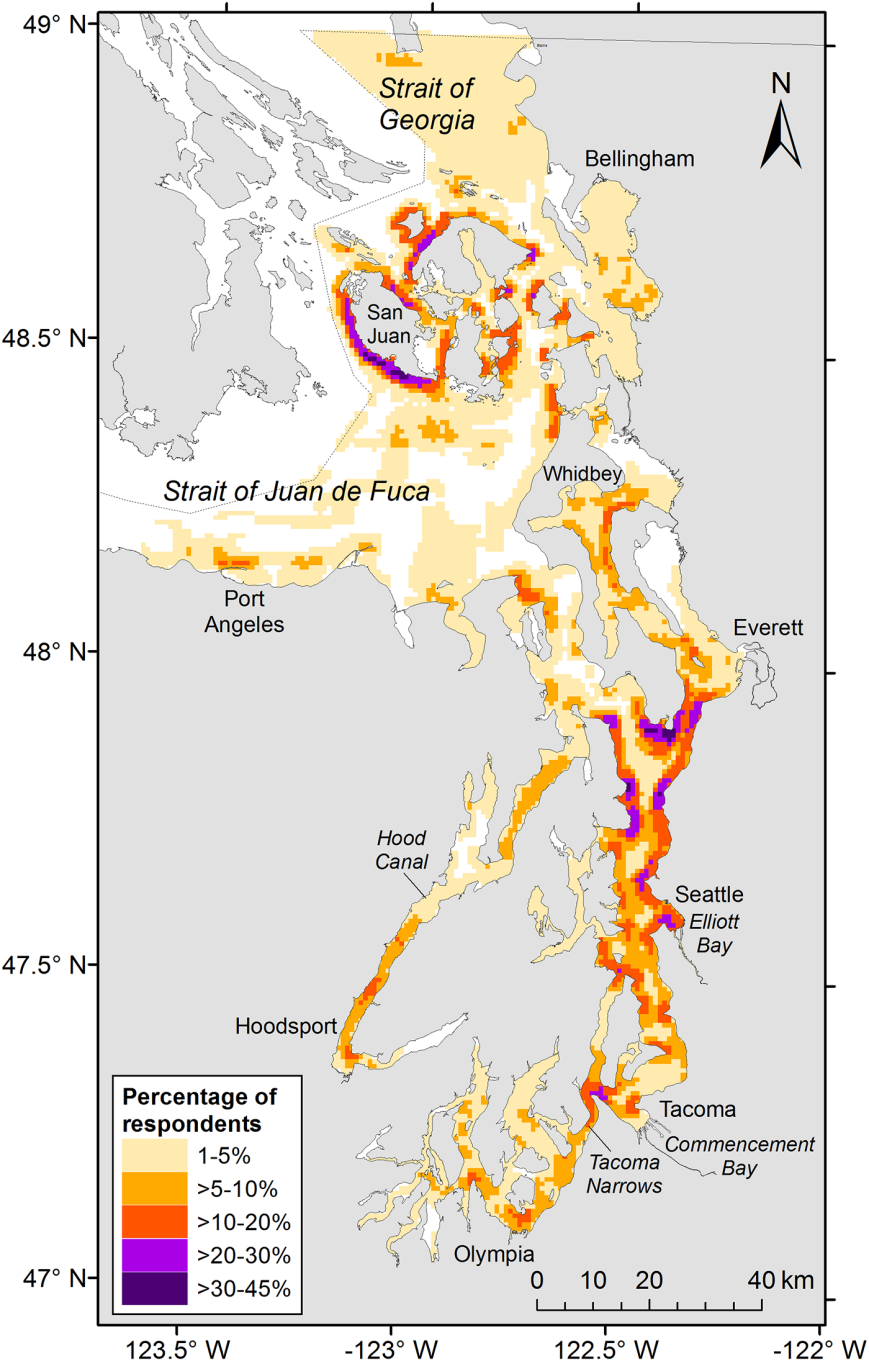
Spatial distribution of recreational fishing for salmon, rockfishes, flatfishes, and crabs in Puget Sound, 1960–2010. The intensity of space use by fishers is shown as the percentage of respondents who fished in each 0.8 x 0.8 km grid cell.

In-person interviews were conducted with 101 individuals residing in twelve counties bordering Puget Sound during 2010 and 2011. For this study, we defined the geographic extent of Puget Sound as the U.S. waters east of approximately 123.765° W, an area encompassing approximately 6400 km^2^ of inland marine waters (Fig 1). We selected respondents with specialized knowledge of marine species acquired from 10 or more years of experience fishing in Puget Sound using a stratified chain referral approach [28] and peer referencing [29]. These non-random sampling methods are commonly used to identify respondents with particular knowledge or expertise (i.e., specialized informants; [28]), rather than produce a representative sample of a larger population. To meet our study objectives, we identified anglers with long-term local knowledge and experience targeting multiple species groups; these individuals may not be representative of the Puget Sound recreational fishing population as a whole. Respondent selection is described in detail by [30] and [31]. The research was reviewed and approved by the University of Washington Human Subjects Division (Certification of Exemption #37330). Respondents provided written informed consent prior to participation in the interviews.

To document spatial patterns of fishing over time, respondents were asked whether their primary fishing locations changed over the time they had fished recreationally for Puget Sound species and when those changes occurred (if any). Respondents were asked to delineate areas in which they targeted particular species during each period. For example, if a respondent indicated a shift in fishing locations in 1990, she or he was then asked to record fishing areas on one chart for the period prior to 1990 and a different chart for the period after. Fishing areas were represented by respondents as polygons hand-drawn on paper maps generated from a Geographic Information System (GIS)-based reference map of Puget Sound projected in the Washington State Plane coordinate system (NAD 1983, HARN, Washington State Plane South FIPS 4602 Feet; http://www.ecy.wa.gov/services/gis/data/data.htm). An appropriate map scale (1:343,062) was determined based on consultation with experienced fishers in Puget Sound. We asked respondents to separately mark target areas for four groups of recreationally harvested species: Pacific salmon (*Oncorhynchus* spp.), rockfishes (*Sebastes* spp.), small-bodied flatfishes (i.e., excluding Pacific halibut; Pleuronectiformes spp.), and crabs (Dungeness crab, *Metacarcinus magister*, and red rock crab, *Cancer productus*). Analyses were limited to fishing maps generated by boat-based anglers (i.e., maps from divers who spearfished were excluded) who had reported target areas for at least one of the four focal species groups (N = 80 respondents; Table 1).

**Table 1:** Number of respondents reporting fishing areas for each species group and decade.

	Species Group				
Decade	Salmon	Rockfish	Flatfish	Crab	All Species
1920	1	0	0	0	1
1930	2	0	0	0	2
1940	4	2	1	0	5
1950	16	7	5	3	16
1960	31	20	8	9	36
1970	46	42	13	24	58
1980	55	46	14	32	69
1990	59	50	12	33	73
2000	57	48	12	36	68
Total	68	62	20	42	80

### Map digitization and analysis

**Fig 2:**
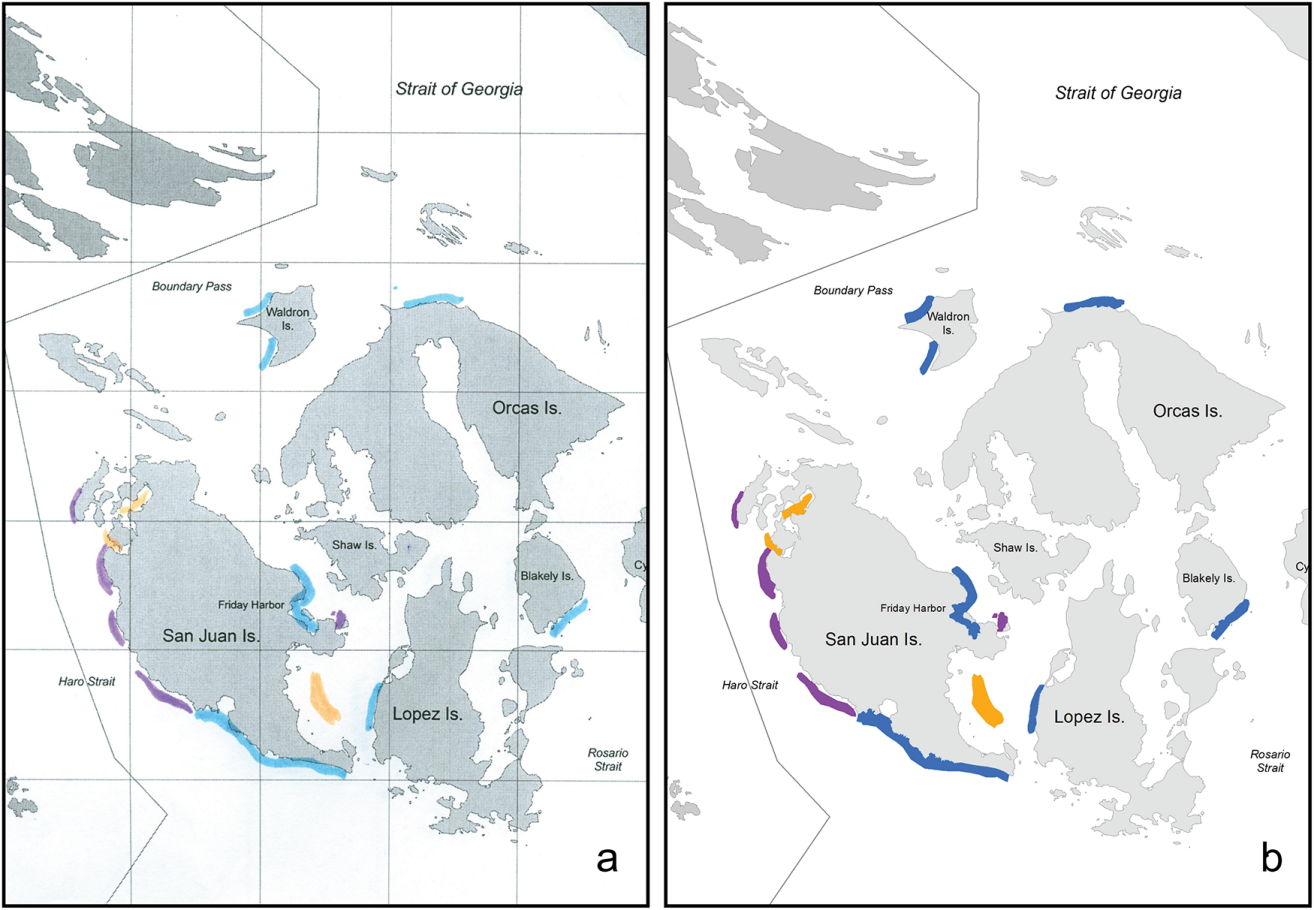
Fishing areas drawn by a recreational fisher (a) and digitized in a Geographic Information System (b). Separate areas were drawn for rockfish (purple), salmon (blue), and crab (orange). A 5-mile grid was overlaid on the paper maps for reference.

Maps generated by fishers were scanned, georeferenced, digitized, and translated into vector polygons in a GIS (ArcGIS 10.0, ESRI, 1990–2010; Fig 2). Scanned images were digitally overlaid on the reference map described above and ground control points were used to accurately georeference the images [18]. Each fishing area marked by a respondent was outlined to create fishing area polygons in a vector-based map layer. Fishing areas were aggregated by interviewee, decade, and targeted species group. The number of respondents who documented fishing areas varied among decades and species groups (Table 1). We excluded the 1920s, 1930s, and 1940s from analyses due to small sample sizes (≤ 5 respondents).

To examine temporal shifts in the size of fishing areas used by individual anglers, we calculated the mean area fished per angler for each decade and species group. Temporal trends in per capita fishing areas were characterized using locally weighted regression (loess; [32]). Loess uses a locally weighted least squares criterion to fit a local linear model at each point, with the fraction of data used in smoothing at each point denoted by smoothing parameter α [33]. We used the default value of 0.75 for α [32]. The distribution of polygon areas for each decade and species group was visualized using boxplots.

To characterize the spatial extent of recreational fishing across all respondents, we quantified core fishing areas by all anglers combined for each decade and species group using kernel density estimation (KDE; [34]). First, fishing area polygons were overlaid on a uniform grid using the spatial join function in ArcGIS 10.0. Due to the smaller average size of rockfish fishing areas, rockfish polygons were overlaid on a half-mile (0.8 km) grid while salmon, crab, and flatfish polygons were overlaid on a one-mile (1.6 km) grid. These grid cell sizes approximated the mode of the distribution of respondents’ polygon areas for each species group. Next, the centroids of grid cells with overlapping polygons provided input point data for KDE. KDE was performed in the Geospatial Modeling Environment (GME) (v.0.7.2.0, http://www.spatialecology.com/gme) using a Gaussian kernel with fixed smoothing [35], and a grid resolution of 1000 ft (305 m). The bandwidth parameter, *h*, was calculated using the multivariate PLUGIN method of [36] using the ks package in R (Rx64, version 2.15.2, http://www.R-project.org/). Unique values of *h* were used for each species group and decade. The PLUGIN method was selected over the *h*_*opt*_ method [34] due the high incidence of coincidental points that occurred in areas with overlapping fishing polygons. Separate analyses were performed for each species group and decade for which data were sufficient to estimate kernel density (rockfish and crab: 1970–2000, salmon and flatfish: 1960–2000).

Core fishing areas were defined by the kernel utilization distribution (KUD), which is interpreted as the area in which there is specified probability that fishing occurred during a given time period. A 50% KUD is more commonly used to delineate core areas in animal movement studies [35]; however, a 25% KUD was used to define core fishing areas because it provided a better visual approximation to the areas of high overlap in use by anglers. Regions of the KUD located on land or in Canadian waters were subtracted from the total area. The spatial distribution of fishing locations for all species combined was visualized by creating a heat map for each decade of the proportion of respondents who fished in each 0.8 x 0.8 km grid cell.

### Anglers’ reasons for behavioral change

We characterized the frequency of respondents demonstrating a shift in fishing locations from the 1950s through the 2000s. First, total area fished for all species combined was calculated for each respondent and decade. Next, for each respondent, the rate of areal change from the 1950s to 2000s was estimated by the slope of a linear regression fit to total area. Slopes were used to classify shifts in fishing area over time as increases (β > 0.1, representing an increase of at least 5 km^2^ from the 1950s to the 2000s), decreases (β < 0.1), or no change (-0.1 ≤ β ≥ 0.1).

During the interviews, respondents were asked to explain their reasons for changing fishing locations or extent over time. In addition, we examined decadal changes in the annual average number of fishing days per angler, as a proxy for fishing effort, from the 1950s to the 2000s. Anglers were asked to report their average number of fishing days per year for all years of participation in the Puget Sound recreational fishery [30]. The trend in fishing effort over time was evaluated by regressing the number of days fished by anglers against decade. A natural log transformation was applied to number of days fished to account for non-normally distributed errors [37].

## Results

**Fig 3:**
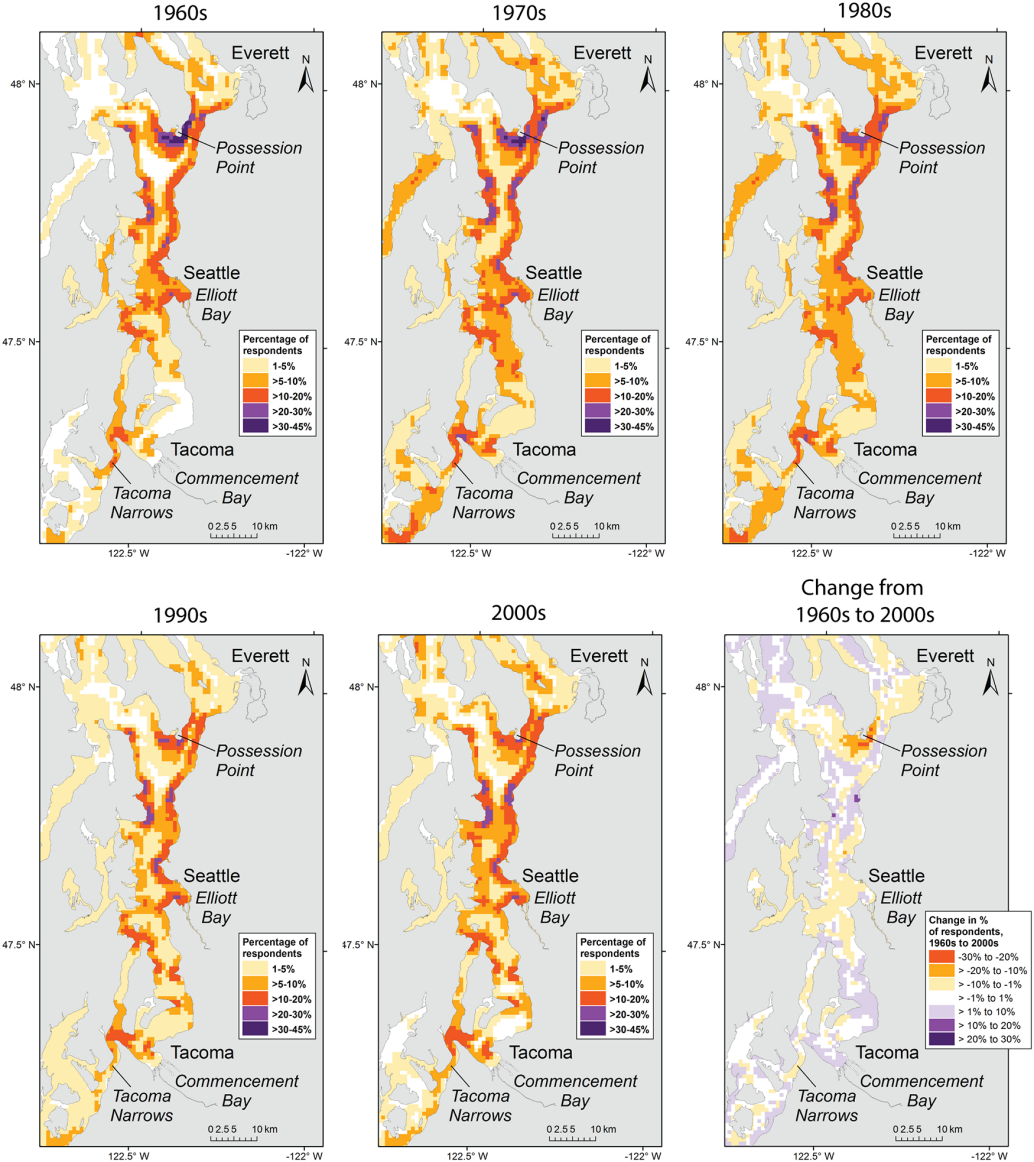
Spatial distribution of recreational fishing for salmon, rockfishes, flatfishes, and crabs in central Puget Sound. Maps for individual decades are shown, from the 1960s to the 2000s. The intensity of space use by fishers is represented as the percentage of respondents who fished in each 0.8 x 0.8 km grid cell. The lower right panel shows the change in percentage of respondents from the 1960s to the 2000s. Positive values indicate that the percentage of respondents fishing in an area was higher in the 2000s than the 1960s.

**Fig 4:**
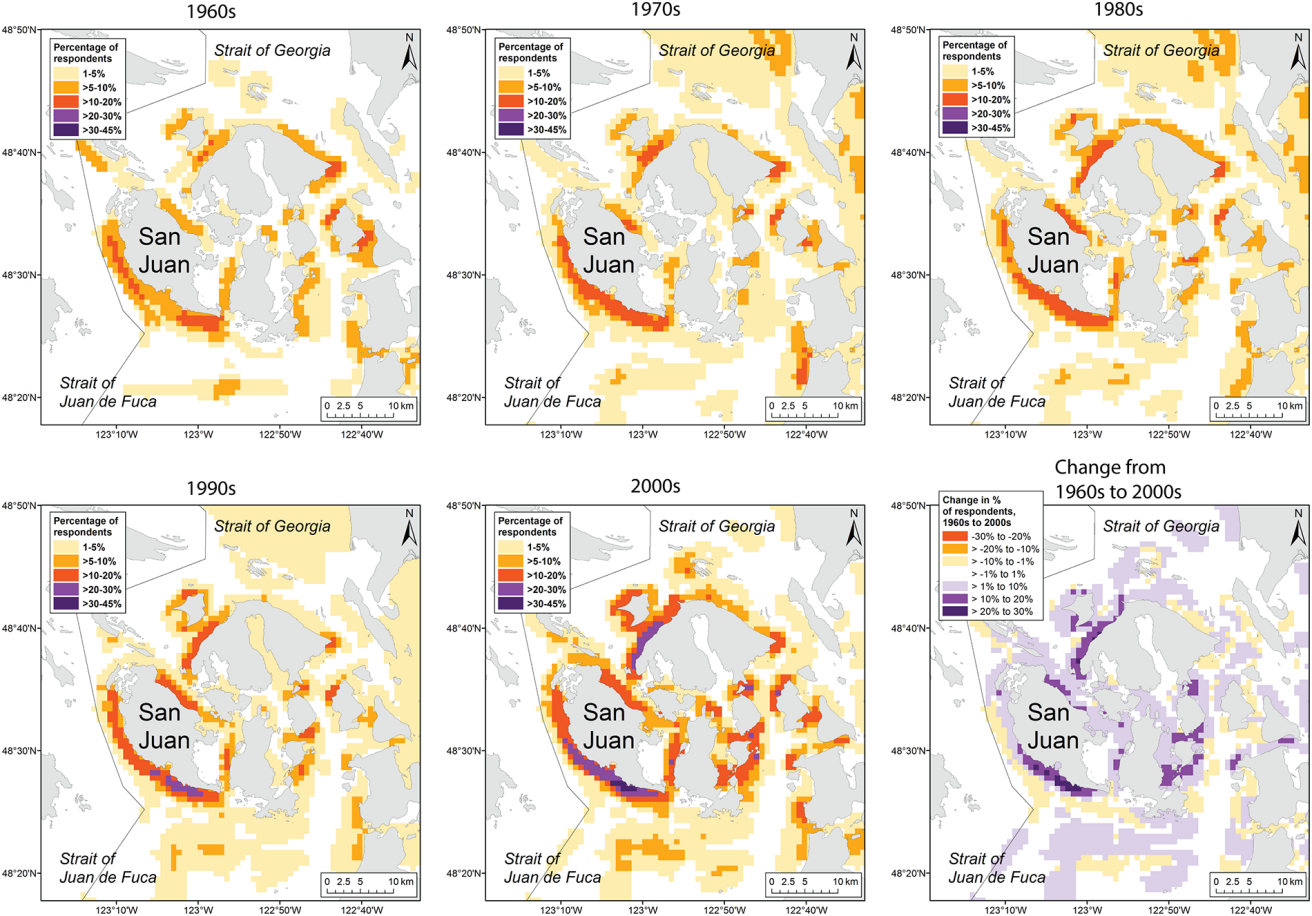
Spatial distribution of recreational fishing for salmon, rockfishes, flatfishes, and crabs in the San Juan Islands. Maps for individual decades are shown, from the 1960s to the 2000s. The intensity of space use by fishers is represented as the percentage of respondents who fished in each 0.8 x 0.8 km grid cell. The lower right panel shows the change in percentage of respondents from the 1960s to the 2000s. Positive values indicate that the percentage of respondents fishing in an area was higher in the 2000s than the 1960s.

Respondents (N = 80) showed extensive use of Puget Sound for recreational fishing since the 1950s (Fig 1). Fishing locations were predominantly concentrated in nearshore regions of Puget Sound, with the highest proportion of respondents reporting use of areas west of San Juan Island and south of Whidbey Island (Fig 1). These relatively high-use areas have persisted over time and, overall, little change was observed in the specific locations anglers have targeted since the 1950s. However, a shift in intensity of use was evident in certain locations, such as the area off Possession Point on south Whidbey Island, for which both the size of the high-use area and proportion of respondents using it declined from the 1960s through the 2000s (Fig 3). The opposite pattern was observed for the San Juan Islands, where the size of the high-use area and proportion of respondents using it increased over the same period (Fig 4). The mean number of days fished by anglers increased from approximately 29 in the 1950s to 49 in the 2000s (*β* = 0.013, *t*(406) = 3.207, *p* = 0.001; Table 2).

**Table 2:** Mean (2 SE) number of days fished per year reported by respondents.

Decade	Mean (2 SE)days per year	N anglers
1950	29.0 (15.0)	31
1960	31.8 (11.5)	53
1970	35.1 (10.2)	71
1980	40.3 (9.4)	80
1990	45.7 (10.5)	86
2000	49.1 (10.9)	87

**Fig 5:**
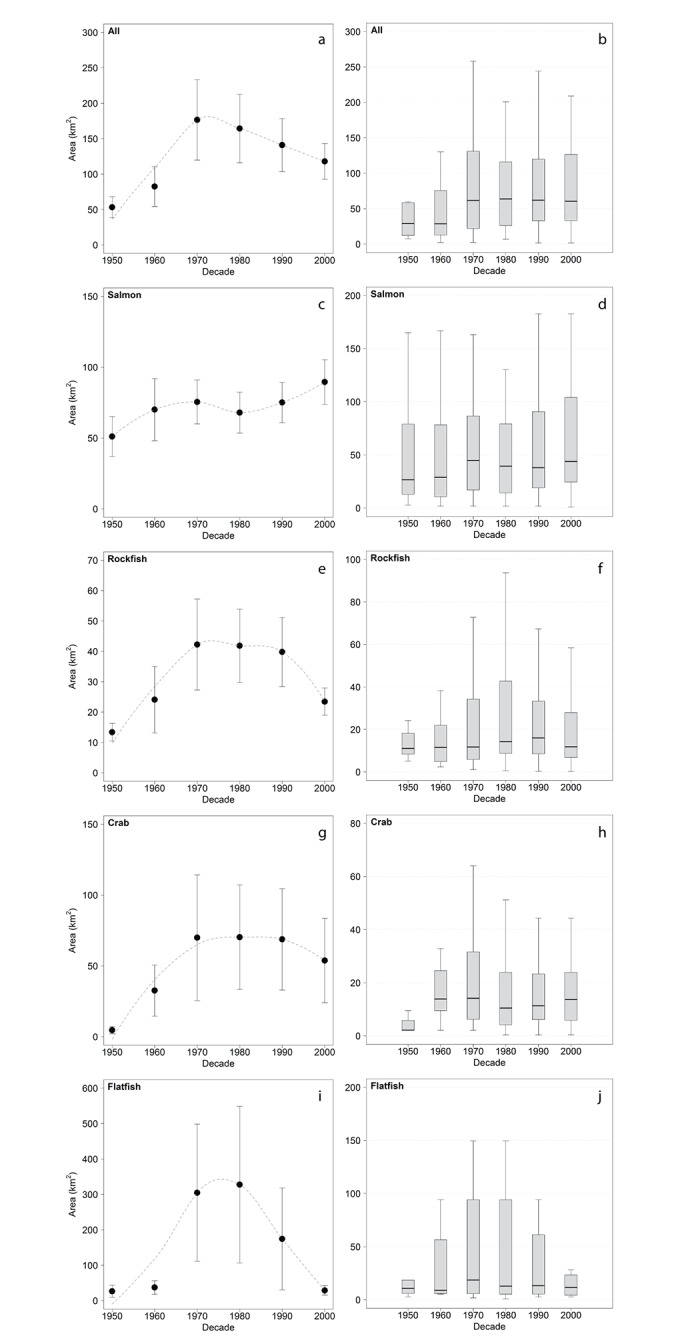
The size of areas fished by individual respondents from the 1950s to the 2000s. Separate plots are shown for all species (a & b), salmon (c & d), rockfishes (e & f), crabs (g & h), and flatfishes (i & j) in Puget Sound. Plots on the left show the mean (±SE) area fished per angler for each decade and fitted locally weighted regression (dashed line). Box plots on the right show median values (solid line) and the interquartile range (box edges). The whiskers extend to the most extreme data point that is no more than 1.5 times the interquartile range from the box.

We characterized changes in the use of space by individual anglers and size of core fishing areas for all respondents combined for salmon, rockfish, crabs, and flatfish since the 1950s. Respondents reported a wide range in the total area targeted for each decade, reflecting substantial individual variation in the extent of anglers’ fishing areas (Fig 5). For all species combined, the mean, median, and range of areas fished by individual anglers increased from the 1950s and 1960s to later decades (Fig 5a and 5b). Three of the four species groups showed a decline in mean area fished from the 1980s to the 2000s (Fig 5). The spatial extent of fishing, as measured by mean area fished per angler and core area used by all respondents, varied among species groups. The mean area per angler and core area targeted for rockfish was smallest (per angler: 34.7 km^2^, core: 597 km^2^), followed by crab (61.6 km^2^, 823 km^2^), salmon (74.7 km^2^, 825 km^2^), and flatfish (178.4 km^2^, 878 km^2^).

**Fig 6:**
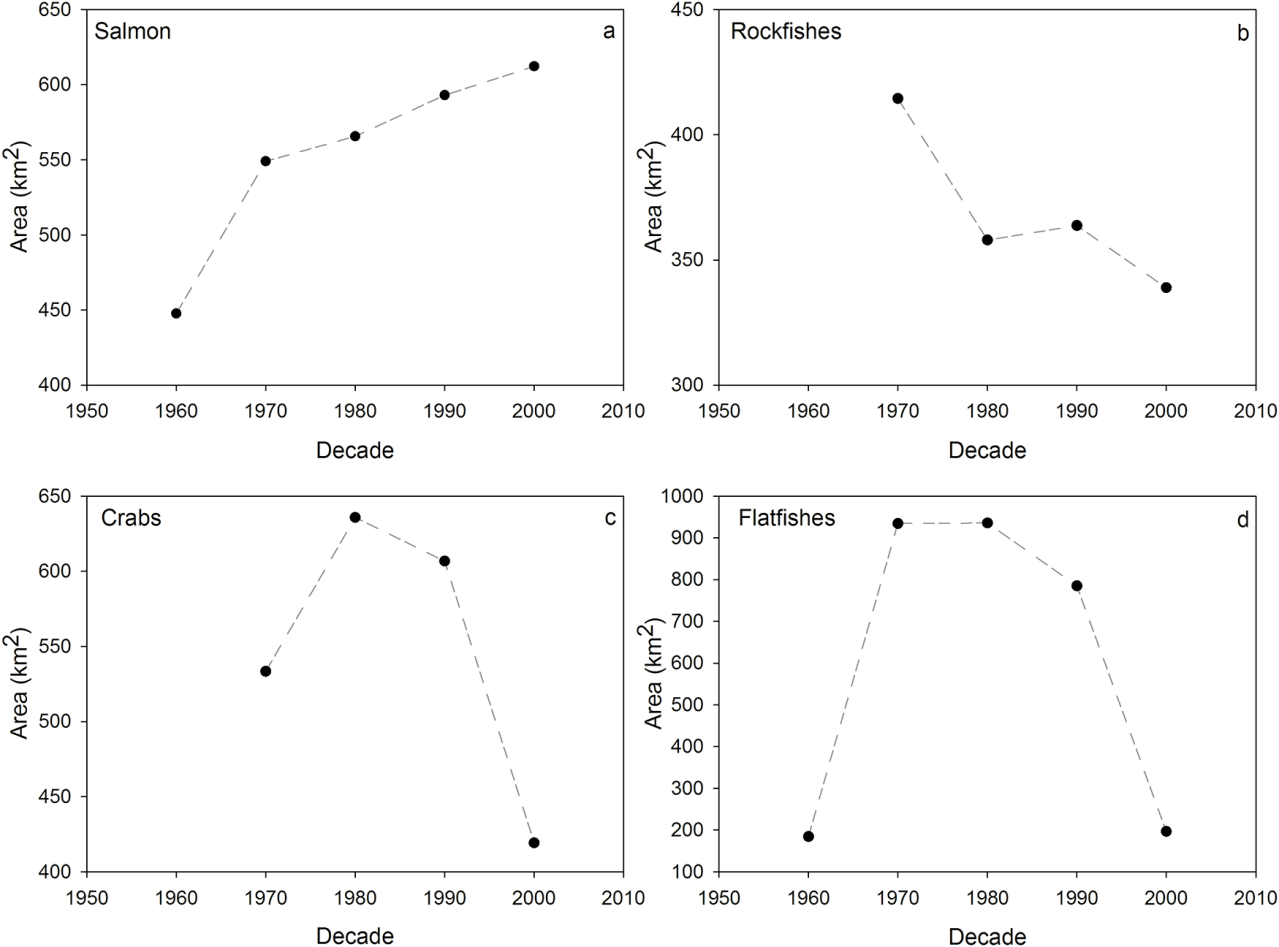
Core fishing areas used by all respondents combined from the 1960s to the 2000s. Separate plots are shown for salmon (a), rockfishes (b), crabs (c), and flatfishes (d) in Puget Sound. Core area is defined as the 25% kernel utilization distribution.

Temporal trends in the mean area fished per angler and core areas for all respondents varied among species groups. The mean area per angler for salmon increased from 51.1 km^2^ in the 1950s to 89.5 km^2^ in the 2000s (Fig 5c), while the range was relatively constant over this period (Fig 5d). The core fishing area for salmon increased from the 1960s to later decades (Fig 6a). For rockfish, the mean area per angler increased from 13.4 km^2^ in the 1950s to a peak of 39.8–42.2 km^2^ during the 1970s-1990s, followed by a decline to 23.4 km^2^ in the 2000s (Fig 5e); rockfish core area showed a similar pattern of decline from the 1970s to the 2000s (Fig 6b). While the median area fished for rockfish showed little change since the 1950s, the range in area fished expanded substantially from the 1950s to the 1980s, followed by a decline in the 1990s and 2000s (Fig 5f). For crabs, the mean area per angler increased from 4.6 km^2^ in the 1950s to 69.9 km^2^ in the 1970s and showed relatively little change in later decades (Fig 5g), while the core area increased from the 1970s to the 1980s, then declined to the lowest level observed in the 2000s (Fig 6c). The range in area fished showed a similar pattern, with an expansion through the 1970s and contraction in later decades (Fig 5h). For flatfish, the mean area per angler increased dramatically from 26.5–37.2 km^2^ in the 1950s-1960s to a peak of 327.6 km^2^ in the 1980s, then declined to 28.8 km^2^ in the 2000s (Fig 5i). The median area fished showed little variation over time but the range peaked in the 1970s and 1980s (Fig 5j). The trend in flatfish core area showed a similar dome-shaped pattern, with core regions more than four times larger in the 1970s-1990s than regions fished in the 1950s and 2000s (Fig 6d).

Of the 80 anglers who provided fishing locations for at least two decades, 28 (35%) indicated no change in the location and/or extent of fishing over time. A total of 29 respondents (36%) showed increases of at least 5 km^2^ in the total area fished in each decade from the 1950s to the 2000s, 18 (23%) showed decreases of at least 5 km^2^, and 5 (6%) showed no change in the extent of area fished but a shift in location. We found support for the hypothesis that anglers’ reported reasons for changes in their fishing areas were related to personal, biological, and regulatory factors. Respondents’ reasons for modifying their fishing behavior were classified into 9 qualitative categories: (1) change in residence, (2) change in work activities, (3) change in recreational interests or activities, (4) gained access to a boat, (5) targeted different species, (6) better fishing or easier access elsewhere, (7) decline in catch rates and/or observed abundance, (8) fishing regulation changes, and (9) no reason provided. A change in residence (16%) and declines in catch rates and/or observed abundance (15%) were indicated as factors leading to shifts in fishing area by the highest number of respondents, while fishing regulation changes were reported as a reason for change by the fewest anglers (5%; Table 3). Sample sizes were too small for a robust statistical analysis of the probability of area increase, decrease, or no change given a particular reason for modifying fishing location or extent. Qualitatively, a higher percentage of respondents reported increases in fishing area compared to those reporting decreases due to a change in residence, better fishing areas elsewhere, a change in recreational interests, gaining access to a boat, and a change in work activities; in contrast, a higher percentage of respondents reported decreases compared to those reporting increases as a result of species declines and fishing regulation changes (Table 3).

**Table 3:** Respondents’ reasons for changes in the geographic location and/or size of fishing areas.

Reason	Decrease	Increase	No change	N	%
No reason provided	5	9	0	14	18%
Change in residence	6	7	0	13	16%
Decline in catch rates and/or observed abundance	7	4	1	12	15%
Targeted different species	5	4	1	10	13%
Better fishing or easier access elsewhere	4	5	1	10	13%
Change in work activities	3	4	2	9	11%
Change in recreational interests or activities	3	5	1	9	11%
Gained access to a boat	2	6	0	8	10%
Fishing regulation changes	3	1	0	4	5%

The total number (N) and percentage (%) of respondents are reported for each reason and categorized by the number who showed a decrease, increase, or no change in the mean size of fishing areas.

## Discussion

Throughout the world’s oceans, most human activity is concentrated in nearshore marine ecosystems that are in proximity to population centers and face numerous cumulative impacts, including habitat degradation, eutrophication, pollution, and fishing [8]. Like most other coastal regions of the world, no part of Puget Sound has been untouched by human activities. Over the past 50 years, the footprint of recreational fishing has spanned the majority of Puget Sound waters (approximately 85% of Washington marine waters east of 123°49.6’ W; Fig 1). This is a conservative estimate, however, as our results are based on fishing locations for just 80 respondents, who account for just 0.04% of Washington resident saltwater sportfishing license holders in 2010–2011 [38]. Our study was limited in its ability to fully assess the spatial impact of recreational fishing on the Puget Sound ecosystem, which would require more detailed, spatially-explicit data on catch and effort (e.g., angler-hours) that are difficult to elicit from retrospective interviews alone. However, the sampling methodology could be modified for future studies to advance participatory mapping as a quantitative tool for recreational fisheries monitoring and assessment. For example, annual surveys of anglers could be conducted to generate more detailed information about areas and days fished during the last year, rather than over multiple decades. Furthermore, it may be possible to survey a larger, more representative population of anglers by conducting interviews at boat launches, e.g., [39].

Together with historical sources, our results suggest that the spatial extent of recreational fishing has likely increased since the 1950s, with the most marked expansion occurring prior to the 1990s. The size of fishing areas increased through the 2000s for salmon but declined after the 1970s and 1980s for rockfish, flatfish, and crab. While our study was not designed to comprehensively assess historical changes in recreational fishing effort, we found that the average number of days fished per year by respondents increased 69% from the 1950s to the 2000s. These findings are consistent with documented trends in recreational bottomfishing activity in Puget Sound [40, 25]. The 1970s and 1980s marked a period of rapid growth, as the availability of motorized vessels and advanced fish-finding technologies allowed anglers to expand their scope beyond localized fishing from piers and boathouses [25]. The number of recreational trips in Puget Sound peaked in the mid-1980s and declined substantially in the 1990s, following reductions in rockfish bag limits intended to reduce mortality on declining rockfish stocks [25]. It is likely that the spatial footprint of recreational angling for bottomfish has been further reduced following 2010 regulations that prohibited rockfish retention and limited bottomfishing depths to shallower than 120 feet [26].

### Species-specific patterns in fishing areas

As expected, there were species-specific differences in the size of areas fished and trends over time. Species-specific trends in the size of core recreational fishing areas, as determined using kernel density estimation, and the mean size of individual anglers’ fishing areas were similar. Corroboration between the two methods suggests that the data reflect real shifts in the size of fishing areas over time, despite substantial variation in the locations and extent of fishing among individuals. On average, target areas were largest for small-bodied flatfishes, intermediate for salmon and crab, and smallest for rockfishes. These patterns likely reflect both the underlying distribution of the species as well as the behavior of fishermen targeting them. Rockfish have small home ranges and strong site-fidelity to high-relief bedrock and kelp forest habitats (“rocky reefs”; [24]). Respondents targeted rockfishes by jigging with hook and line in proximity to rocky reefs; therefore, fishing areas for rockfishes were small and reflected the patchy distribution of rocky reefs in Puget Sound [41, 42]. In contrast, salmon are mobile, pelagic species that are commonly targeted by trolling with artificial lures, a fishing technique that allows for relatively rapid coverage of large areas and multiple depths. Salmon were the only target species group that showed an overall increase in the size of fishing areas over time, with core areas expanding 37% from the 1960s to the 2000s and mean size of individual areas increasing 75% from the 1950s to the 2000s. Flatfishes, crab, and rockfishes showed a dome-shaped trend in the size of individual fishing areas, with a peak in both mean size and variance of fishing areas during the 1970s and 1980s. This downward trend in mean size of individual areas from the 1970s onward was reflected by declines in core area size over the same period.

### Stability in locations of core fishing areas

While recreational fishing has taken place throughout most, if not all, of Puget Sound, the heaviest recreational use was limited to relatively small areas of central Puget Sound and the San Juan Islands. As a whole, the anglers we interviewed are still fishing in the same places they were decades ago, with relatively little change in specific locations. Despite widespread declines in salmon and bottomfish populations during the second half of the 20^th^ century [43, 25], locations of core fishing areas at the south end of Whidbey Island and the west side of San Juan Island remained stable from the 1950s through the 2000s. Possession Bar, at the south end of Whidbey, has long been viewed as a productive area by fishermen and was documented as an important commercial fishing ground for salmon as early as 1889 [44]. The San Juan Islands and Possession Bar were identified as areas heavily utilized by recreational fishermen for bottomfish, including rockfishes, in maps published by the Washington Department of Natural Resources in 1972 [45, 46]. Core fishing areas coincide with areas where rockfishes, salmon, and flatfishes frequently occurred, according to an atlas published by University of Washington researchers in 1980, the most comprehensive source of information on the geographic distribution of fishes in Puget Sound [47]. The atlas compiled all available data up to 1972 on the spatial occurrence of Puget Sound fishes from the published literature and research records at the University of Washington and government agencies [47].

The spatial stability of core fishing locations is not necessarily indicative of the sustainability of resources harvested there. In small-scale fisheries, the location of fishing spots may remain stable even when resources are scarce because of the social, cultural, and economic factors that influence where people fish, e.g., [48, 19]. For example, artisanal fishers in Brazil showed use of the same areas for three decades, despite increased urbanization and declines in fish abundance and diversity in some locations [48]. Temporal stability in fishing spots stemmed from a tradition of people fishing close to home, due to low-powered vessels that limited travel distance and an informal division of fishing areas among communities [49, 48]. Knowledge of where people fish can help reveal perceptual differences between fishers and scientists about the abundance and distribution of harvested species that can lead to conflict in natural resource management. For example, catch-per-unit-effort may remain stable even with a declining fish population because of fish aggregating behavior [50] and fishers’ ability to target these aggregations (e.g., *Paralabrax* spp. fisheries in California, [51]). Termed hyperstability [52], this can lead to discordant perceptions of stock condition between fishers who experience stable or increasing catch rates and scientists who see evidence of declines from fishery-independent data.

### Shifts in fishing areas and reasons for change

In Puget Sound, while the location of core areas remained consistent over a 50 year period, the size of those areas and intensity of use changed over time. For example, the percentage of respondents fishing in Central Puget Sound declined from the 1960s through the 2000s, and the size of the high-use areas near Possession Bar contracted over that period (Fig 3). Conversely, the San Juan Islands showed an expansion of core areas and an increase in the percentage of respondents fishing there (Fig 4). Whether this pattern indicates a northerly shift in the recreational fishery since the 1960s or is driven by changes in fishing behavior or place of residence over the lifetime of the respondents is unclear. Commercial fishers in Newfoundland showed increases in engine power, vessel capacity, and fishing gear ownership over the course of their careers, as increased income allowed for greater financial investment in the fishery [13]. Likewise, changes in fishing capacity over the lifetime of individual anglers may underlie spatial shifts in fishing intensity in Puget Sound. For example, some respondents explained increases in fishing effort and/or spatial extent of fishing as the result of a shift from shore-based fishing as children to boat-based fishing as young adults, acquisition of larger, more powerful vessels later in their careers, or increased leisure time following retirement. Because of these age-related shifts in fishing behavior, temporal trends in use of specific fishing areas by the respondent group may not be generalizable to the larger population of recreational anglers in Puget Sound.

In recreational fisheries, where people fish and why they fish there is tied strongly to personal reasons. Characterizing this individual variation is important for understanding recreational fishery dynamics, which may be harder to predict than for commercial fishers, due to a greater diversity of motivations and behaviors. This individual variation is reflected in the large distribution in mean size of fishing areas among respondents and the wide range of reasons they provided for shifts in mean size and/or geographic location of fishing areas over time. While the majority of respondents (65%) reported temporal shifts in the mean size and/or geographic location of their fishing areas, there was no dominant mechanism to explain changes over time. Changes in residence and declines in target species were the most frequently cited reasons for shifts in where people fished. Where people lived primarily affected the location rather than the size of their fishing areas, because respondents tended to fish close to home. Respondents sought additional or alternative fishing areas in response to species declines, resulting in either increases or decreases in the mean size of fishing areas. Shifts in attributes of fishing areas were least tied to fishery regulation changes despite the implementation of at least 25 marine preserves and conservation areas that are permanently or periodically closed to fishing for salmon and bottomfish since 1970 [53]. In this and a related study of recreational anglers in Puget Sound, respondents explained that they stopped targeting rockfish in advance of conservation measures imposed by Washington State in response to their personal observations of species declines and growing understanding of rockfish vulnerability to fishing [39]. Given that local ecological knowledge of fishers is often the only source of information on species abundance and distribution, as is the case for some rockfishes in Puget Sound [31], restricted access to fishing grounds can result in a loss of valuable place-based information about the environment.

## Conclusions

Knowledge about the spatial distribution of fishing activities can inform place-based management that balances species conservation with opportunities for recreation and subsistence. For example, participatory mapping has contributed to the development of spatial management approaches that promote species recovery while minimizing socioeconomic impacts on stakeholders, e.g., [54, 55, 10]. This study provides the most comprehensive documentation to date on historical fishing locations for rockfish in Puget Sound, which was identified by NOAA as a key information need for ongoing recovery planning of ESA-listed rockfishes. Spatial information about rockfish fishing activities can be used to make inferences about the historical distribution of productive rockfish habitat. Knowledge of fishing locations is also critical for identifying areas of historical importance to anglers, interpreting their local knowledge about rockfish populations (e.g., [31]), and engaging them in conservation efforts. Where people fish affects their perceptions of factors threatening recovery of ESA-listed rockfishes and preferences for rockfish recovery measures [39]. Gaining the necessary public support to meet rockfish recovery goals may, therefore, depend on developing conservation strategies that resonate with anglers’ place-based experience and local knowledge of the marine environment.

## Supporting Information

S1 Appendix Questionnaire. After the respondent provided informed consent, the interviewer administered the questionnaire verbally and took written notes. If the respondent provided consent to audio record the interview, the interviewer’s notes were checked against audio recordings.
(DOC)

S1 Dataset Per capita fishing area. Area fished per angler for each decade and species group. Data include: respondent identification number (ResID), species group (Sp.Grp), decade corresponding to respondent’s mapped fishing areas (Decade), and total area (km^2^) fished by the respondent for a given species group and decade (Total.Area.sq.km).
(CSV)

S2 Dataset Spatial distribution of recreational anglers for all species, 1960–2000. The total number of anglers (Frequency) and proportion of respondents (Proportion) who fished in each 0.8 x 0.8 km grid cell is provided. CELLID is a unique identification number for each grid cell. The values MinX, MaxX, MinY, and MaxY designate the vertices of each grid cell; CenterX and CenterY are the coordinates for the centroid of each grid cell. These data can be used to create a map of the proportion of respondents fishing in each 0.8 x 0.8 km grid cell within Puget Sound (i.e., Fig 1). Map projection: NAD 1983, HARN, Washington State Plane South FIPS 4602 Feet.
(CSV)
