# Supplementary material for: Historical Patterns and Drivers of Spatial Changes in Recreational Fishing Activity in Puget Sound, Washington
Source: PLoS One. 2016 Apr 7;11(4):e0152190. doi: 10.1371/journal.pone.0152190 (PMC4824406; doi:10.1371/journal.pone.0152190)
Supplement: S1 Appendix — After the respondent provided informed consent, the interviewer administered the questionnaire verbally and took written notes. If the respondent provided consent to audio record the interview, the interviewer’s notes were checked against audio recordings. (DOC) [file pone.0152190.s001.doc]

**S1 Appendix. Questionnaire.** After the respondent provided informed consent, the interviewer administered the questionnaire verbally and took written notes. If the respondent provided consent to audio record the interview, the interviewer’s notes were checked against audio recordings.

**1. Experience and personal history**

In what ways have you gained experience with **marine species in Puget Sound**? Mark more than one if applicable.

□ Commercial fishing

Target species: ________________________________________________________________

Methods / gear type(s) used: _____________________________________________________

Years (e.g., 1990-2005) and approx. number of days per year: *If this changed over time, ask respondent to specify average number of days per year for each group of years individually.*

____________________________________________________________________________

____________________________________________________________________________

□ Recreational fishing

Target species: ________________________________________________________________

Methods / gear type(s) used: _____________________________________________________

Years (e.g., 1990-2005) and approx. number of days per year: *If this changed over time, ask respondent to specify average number of days per year for each group of years individually.*

____________________________________________________________________________

____________________________________________________________________________

□ Diving (non-harvest)

Target species: ________________________________________________________________

Years (e.g., 1990-2005) and approx. number of days per year: *If this changed over time, ask respondent to specify average number of days per year for each group of years individually.*

____________________________________________________________________________

____________________________________________________________________________

□ Research

Target species: ________________________________________________________________

Sampling method(s) used: _______________________________________________________

Years (e.g., 1990-2005) and approx. number of days per year: *If this changed over time, ask respondent to specify average number of days per year for each group of years individually.*

___________________________________________________________________________

___________________________________________________________________________

□ Other, please specify

Target species: ________________________________________________________________

Method(s) used: _______________________________________________________________

Years (e.g., 1990-2005) and approx. number of days per year: *If this changed over time, ask respondent to specify average number of days per year for each group of years individually.*

____________________________________________________________________________

____________________________________________________________________________

*continued next page*

**2. Fishing locations**

*Interviewees are given a paper chart for each decade in which they had experience with Puget Sound marine species and asked to draw the areas in which they fished, dove or conducted research during that period for each species group. Each species group is marked in a different color, e.g., salmon (blue), rockfishes (purple), crabs (orange), flatfishes (green). If temporal changes in the location or geographic extent of fishing are indicated, the respondent is asked* ***why*** *these spatial changes occurred.*

Notes:

**3. Demographic information**

*Respondent completes this section.*

1. In what city or town do you live?

2. In what year were you born?

3. What is your sex?

□ Male □ Female

4. What is your race? Mark one or more boxes.

□ White □ Black or African American

□ Asian □ Native Hawaiian or other Pacific Islander

□ American Indian or Alaska Native – Name of principal tribe: __________________________

____________________________________________________

□ Other, please specify:__________________________________________________________

5. Are you Hispanic or Latino?

□ No □ Yes

Can you suggest anyone else to contact for an interview?
